# The global Multidimensional Poverty Index: Harmonised level estimates and their changes over time

**DOI:** 10.1038/s41597-024-04269-x

**Published:** 2025-01-26

**Authors:** Nicolai Suppa, Usha Kanagaratnam

**Affiliations:** 1 Serra Húnter Fellow, Department of Econometrics, Statistics and Applied Economics, University of Barcelona, 08034 Barcelona, Spain; 2 Oxford Poverty and Human Development Initiative (OPHI), Oxford Department of International Development, Oxford University, Oxford, OX1 3TB United Kingdom

**Keywords:** Interdisciplinary studies, Developing world, Economics, Sociology, Education

## Abstract

This paper describes the database *The global Multidimensional Poverty Index (MPI): Harmonised level estimates and their changes over time*. The global MPI is an international poverty measure based on ten deprivation indicators in three dimensions: health, education, and living standards. The database contains estimates for the MPI itself (the adjusted headcount ratio); related partial indices such as headcount ratio, intensity, indicator-specific indices, and several auxiliary statistics; and changes over time for most quantities. For this database, all deprivation indicators have been harmonised over time. Our database covers estimates for 84 countries and 814 subnational regions for up to four points of observation. The estimates are based on 211 individual survey datasets, provided primarily by the Demographic Health Surveys (DHS) and the Multiple Indicator Cluster Surveys (MICS). Combining information about different dimensions of human wellbeing, the global MPI inherently invites interdisciplinary research.

## Background & Summary

Despite substantial progress in recent decades, poverty is one of the most pressing problems of our time. It is widely agreed that both poverty and wellbeing *are* multidimensional concepts. For example, the Human Development Index (HDI), first published in 1990, draws on information from health, education, and income. The Millennium Development Goals (MDGs) and their successors, the Sustainable Development Goals (SDGs), include targets such as zero hunger, universal education, and access to clean water and sanitation. At the same time, research extended conventional poverty measurement^1,2^ to multidimensional settings^3–6^. Measures of multidimensional poverty allow us to identify overlapping deprivation at the individual level and are therefore fundamentally different from both composite indices, such as the HDI, and (unidimensional) monetary poverty measures. In his last book, Sir Tony Atkinson set forth the paradigm of triangulating across (i) monetary and multidimensional poverty measures and (ii) international and national measures to better understand poverty dynamics and their drivers^7^.

In this paper, we document the global Multidimensional Poverty Index (MPI) data, or more specifically, its harmonised level estimates and related changes over time^8^, which are essential to understanding and reinforcing poverty reductions. The global MPI was developed by Sabina Alkire and Maria Emma Santos, two scholars from the Oxford Poverty and Human Development Initiative (OPHI)^9,10^, in collaboration with the Human Development Report Office (HDRO) at the United Nations Development Programme (UNDP), as an internationally comparable measure of acute poverty. The global MPI comprises ten indicators organised in three dimensions (health, education, and living standards). Technically, the global MPI relies on the Alkire–Foster method^6^, and conceptually, it is based on the capability approach^11–14^. While indicator decisions were informed by the MDGs, they were also data constrained. In 2018, five of the ten indicators were revised to better align with the SDGs^15–17^. The global MPI is estimated using comprehensive household survey data, provided primarily by the Demographic and Health Surveys (DHS) and the Multiple Indicator Cluster Surveys (MICS).

Since 2010, the global MPI has been computed and published annually based on the most recent dataset for each country. The related changes over time, which involve indicator harmonisation, have so far been published only infrequently, often for only a few countries^18,19^, or for only two time periods^20^, or for individual countries^21,22^. Since 2021, however, harmonised level estimates and their changes over time have been computed annually, the objective being to provide estimates that are comparable across countries and time. Further datasets have been added where possible, and all estimates are now available in a single database. Although the database was first published in 2021, this paper is the first description of it. We focus on the 2023 release of the database. The databases may be distinctively different each year, as additional survey datasets are harmonised for countries where updated surveys are available. The inclusion of additional harmonised surveys for a country can result in differences in the changes over time estimates for the country compared with the previous release of the database, due to decisions about indicator harmonisation. The latest release of this database is based on 211 individual survey datasets from 84 countries for 2–4 points of time and provides estimates for 814 subnational regions from 77 countries. The database provides estimates for urban and rural areas and age groups. All estimates are accompanied by standard errors and confidence intervals reflecting sampling errors, which cannot be taken for granted in poverty statistics^23^.

The estimates are organised into two well-structured files, one for levels and one for changes, to provide easy, transparent, and user-friendly access. The potential use of our database includes several distinct forms of analysis, which help to further the knowledge on both global and regional poverty. First, research can directly scrutinise global multidimensional poverty, its changes over time, related projections or simulations^18,22,24,25^, which now requires much less time and specialist knowledge. Second, future research may also explore the connection between multidimensional poverty and other concepts through merging additional data such as environmental indicators, similar to previous work on governance and conflict, for instance^26,27^. Third, research may explore global poverty also within the paradigm laid out by Sir Tony Atkinson via triangulation, i.e., jointly from monetary and multidimensional angles, and together with national poverty estimates. Finally, multidimensional poverty measurement inherently goes beyond disciplinary borders, involving research in health and education, among others. Indeed, recent research suggests a novel analysis of deprivation interlinkages that is potentially relevant for both researchers and policymakers in different fields^28^.

## Methods

The global MPI relies on the Alkire–Foster method. The method requires selecting dimensions and indicators, applying deprivation cutoffs for each indicator, setting relative indicator weights, and defining a cross-dimensional poverty cutoff to identify those who are multidimensionally poor. Estimates are obtained from representative household survey data. We discuss each element in turn, and detail relevant decisions and policies along the way.

### The Alkire–Foster method

To facilitate explanations of the estimates distributed within this database, this section first presents the Alkire–Foster method^6^, which underlies the global MPI computations. A more comprehensive textbook presentation is also available^29^.

Consider for $t=\mathrm{1,}\ldots ,T$ time periods the populations of $i=\mathrm{1,}\ldots ,{N}_{t}$ individuals with $j=\mathrm{1,}\ldots ,D$ achievements in different dimensions of wellbeing. These achievements are denoted by ${y}_{ijt}\in {{\mathbb{R}}}^{+}$. An individual is deprived in $j$ if the respective achievement falls short of the deprivation threshold ${z}_{j}$, or, formally, ${d}_{ijt}={\mathbb{I}}({y}_{ijt} < {z}_{j})$, with ${\mathbb{I}}(\cdot )$ being the indicator function. Let ${w}_{j}\in \mathrm{(0,1)}$ with ${\sum }_{j}{w}_{j}=1$ denote the set of normative weights for all deprivation indicators. The deprivation score of individual $i$ at time $t$ is ${c}_{it}={\sum }_{j}{w}_{j}{d}_{ijt}$ with ${c}_{it}\in \mathrm{(0,1)}$ and reflects the degree of overlapping deprivation experienced by person $i$.

Persons with a critically high deprivation score are considered poor; formally, ${{\rm{poor}}}_{it}={\mathbb{I}}({c}_{it}\ge k)$, where $k\in \mathrm{(0,1)}$ is the poverty cutoff. Let ${Q}_{t}=\{i|poo{r}_{it}=1\}$ be the set of all poor people in period $t$, and ${q}_{t}$ their number. The proportion of the population that is poor, known as the headcount ratio or incidence of poverty, can be obtained as ${H}_{t}={q}_{t}/{N}_{t}$. The poverty intensity, which reports the average deprivation among the poor, is ${A}_{t}=\frac{1}{{q}_{t}}{\sum }_{i\in {Q}_{t}}{c}_{it}$. The adjusted headcount ratio, denoted as $MPI$ or $M$, is ${M}_{t}={H}_{t}\times {A}_{t}$. The deprivation-specific uncensored headcount ratios report the proportion of people who are deprived in a particular indicator $j$ and can be written as ${h}_{jt}=\frac{1}{{N}_{t}}{\sum }_{i}{d}_{ijt}$. Censored headcount ratios report the proportion of people who are both poor and deprived in indicator $j$ and can be written as ${\underline{h}}_{jt}=\frac{1}{{N}_{t}}{\sum }_{i\in {Q}_{t}}{d}_{ijt}$.

The adjusted headcount ratio satisfies several useful properties^6^ including *dimensional breakdown*, which allows computation of the adjusted headcount ratio as ${M}_{t}={\sum }_{j}{w}_{j}{\underline{h}}_{jt}$. The contributions of indicators can be reported in absolute terms, as ${w}_{j}{\underline{h}}_{jt}$ in units of $M$, or in relative terms, as ${w}_{j}{\underline{h}}_{jt}/{M}_{t}\times 100$, i.e., as a percentage of $M$. Disaggregation by subpopulations is also often instructive. More specifically, if the population can be divided into $l=,1\ldots ,L$ mutually exclusive groups of size ${N}_{lt}$, the above-mentioned (sub)indices can also be expressed as population weighted averages, e.g., ${H}_{t}={\sum }_{l}\frac{{N}_{lt}}{{N}_{t}}{H}_{lt}$.

Finally, changes over time can be computed for each of the (sub)indices either in absolute terms (e.g., $\varDelta {M}_{t}={M}_{t}-{M}_{t-1}$) or in relative terms (e.g., $\delta {M}_{t}=\varDelta {M}_{t}/{M}_{t-1}$). To compare changes over different observation periods, it is often useful to look at annualised changes. Annualised absolute changes can be computed as $\overline{\varDelta }{M}_{t}=\frac{\varDelta {M}_{t}}{\varDelta t}$, where $\varDelta t$ is the difference in years between the final period and the initial period; annualised relative changes can be computed as $\overline{\delta }M=\left[{\left(\frac{{M}_{t}}{{M}_{t-1}}\right)}^{\frac{1}{\varDelta t}}-1\right]\times 100$.

### Deprivation indicators, weights, and cutoffs

The global MPI relies on ten deprivation indicators which capture shortfalls in three dimensions: health (*nutrition* and *child mortality*), education (*years of schooling* and *school attendance*), and living standards (*cooking fuel*, *sanitation*, *drinking water*, *electricity*, *housing*, and *assets*). Table 1 provides further details on each of the deprivation indicators. While indicator decisions were informed by the MDGs, they were also data constrained. Previous research–well before the first estimation of the global MPI in 2010–found insufficient data, so dimensions of poverty were missed^30^. Considerations during the 2018 revision for a better alignment of the deprivation indicators with the SDGs revealed that such data constraints persist for a substantial share of the countries^15^. As a consequence, only five indicators were eventually revised, although 33 alternative or new indicators were originally considered. In 2020, the construction of the drinking water indicator was also updated, following the reclassification of safe drinking water sources by the Joint Monitoring Programme for Water Supply Sanitation and Hygiene^31^.

**Table 1:** Global MPI indicator definitions and weights.

Dimension of Poverty	Indicator	Deprived if living in a household where…	SDG area	Weight
Health	Nutrition	Any eligible person is *undernourished*^1^.	SDG 2	$\frac{1}{6}$
	Child mortality	A child *under 18* has *died* in the household in the five-year period preceding the survey^2^.	SDG 3	$\frac{1}{6}$
Education	Years of schooling	*No* eligible household member has completed *six years* of *schooling*^3^.	SDG 4	$\frac{1}{6}$
	School attendance	Any school-aged child3 is *not attending* school *up to* the age at which he/she would .complete *class 8*^4^.	SDG 4	$\frac{1}{6}$
Living				
Standards	Cooking fuel	A household cooks using *solid fuel*, such as dung, agricultural crop, shrubs, wood, charcoal or coal^5^.	SDG 7	$\frac{1}{18}$
	Sanitation	The household has *unimproved* or *no* sanitation *facility* or it is improved but *shared* with other households^6^.	SDG 6	$\frac{1}{18}$
	Drinking water	The household’s source of *drinking water* is *not safe* or safe drinking water is a *30-minute walk* or *longer walk* from home, roundtrip^7^.	SDG 6	$\frac{1}{18}$
	Electricity	The household has *no electricity*^8^.	SDG 7	$\frac{1}{18}$
	Housing	The household has *inadequate* housing materials in *any* of the three components: *floor*, *roof*, or *walls*^9^.	SDG 11	$\frac{1}{18}$
	Assets	The household does *not own more than one* of these *assets*: radio, TV, telephone, computer, animal cart, bicycle, motorbike, or refrigerator, and does not own a car or truck.	SDG 1	$\frac{1}{18}$

**Notes:** For more details, including country-specific decisions, see the underlying methodological note^32^. The global MPI is related to the following SDGs: No Poverty (SDG 1), Zero Hunger (SDG 2), Health & Well-being (SDG 3), Quality Education (SDG 4), Clean Water & Sanitation (SDG 6), Affordable & Clean Energy (SDG 7), Sustainable Cities & Communities (SDG 11).

^1^Children under 5 years (60 months and younger) are considered undernourished if their z-score of either height-for-age (stunting) or weight-for-age (underweight) is below minus two standard deviations from the median of the reference population. Children 5–19 years (61–228 months) are identified as deprived if their age-specific BMI cutoff is below minus two standard deviations. Adults older than 19 to 70 years (229–840 months) are considered undernourished if their Body Mass Index (BMI) is below 18.5 kg/m^2^. ^2^The child mortality indicator of the global MPI is based on birth history data provided by mothers aged 15–49. In most surveys, men have provided information on occurrence of child mortality as well but this lacks the date of birth and death of the child. Hence, the indicator is constructed solely from mothers. However, if the data from the mother is missing, and if the male in the household reported no child mortality, then we identify no occurrence of child mortality in the household. ^3^If all individuals in the household are in an age group where they should have formally completed six or more years of schooling, but none have this achievement, then the household is deprived. However, if any individuals aged 10 years and older reported six years or more of schooling, the household is not deprived. ^4^Data source for the age children start compulsory primary school: DHS or MICS survey reports; or http://data.uis.unesco.org/. ^5^If survey report uses other definitions of solid fuel, we follow the survey report. ^6^A household is considered non-deprived in sanitation if it has some type of flush toilet or latrine, or ventilated improved pit or composting toilet, provided that they are not shared. If the survey report uses other definitions of improved sanitation, we follow the survey report. ^7^A household is considered non-deprived in drinking water if the water source is any of the following types: piped water, public tap, borehole or pump, protected well, protected spring, or rainwater. It must also be within a 30-minute walk, round trip. If the survey report uses other definitions of improved drinking water, we follow the survey report. ^8^A small number of countries do not collect data on electricity because of 100% coverage. In such cases, we identify all households in the country as non-deprived in electricity. ^9^Deprived if floor is made of natural materials (mud/clay/earth, sand or dung) or if dwelling has no roof or walls or if either the roof or walls are constructed using natural or rudimentary materials such as such as carton, plastic/ polythene sheeting, bamboo with mud/stone with mud, loosely packed stones, uncovered adobe, raw/reused wood, plywood, cardboard, unburnt brick or canvas/tent. The definition of natural and rudimentary materials follows the classification used in country-specific DHS or MICS questionnaires..

Technically, the deprivation indicators are constructed at the household level using both household and individual information (see Table 1). Some deprivation indicators, such as those for living standards, use only household-level data. However, health and education indicators make use of individual-level data. For instance, if a school-age child is not attending school, the entire household is considered deprived in school attendance. Likewise, if at least one household member completed six years of schooling, all household members are considered non-deprived in years of schooling.

Some households lack an eligible population, that is, individuals for whom the achievement has been measured. In these cases, the entire household is identified as non-deprived in the particular indicator. For example, if a household has no eligible members for an anthropometric measurement (e.g., children under five years old), the entire household is identified as non-deprived in nutrition. Similarly, the child mortality indicator is constructed using birth history data that are usually collected from women in the reproductive age group (15–49 years). If a household lacks women in the reproductive age group, then all members of this households are identified as non-deprived in child mortality. Finally, throughout the construction of all the deprivation indicators, we rely only on usual household members and thus exclude data from non-usual household members. This approach ensures comparability between survey datasets (some datasets only provide information for usual household members) and reduces fluctuations in deprivations due to occasional visitors.

In addition to the deprivation indicators themselves, Table 1 also shows the respective weights, which follow from an equal-nested weighting structure, i.e., (i) all dimensions are weighted equally at one-third and (ii) all deprivation indicators are weighted equally within each dimension. Similarly to the deprivation indicators, poverty is established at the household level, i.e., the unit of identification is the household. The poverty cutoff for the global MPI is set to $k=\frac{1}{3}$, implying that a household is considered poor if it suffers from $\frac{1}{3}$ or more of the maximum possible weighted deprivations. A person is identified as vulnerable to poverty if they are deprived in 20–33.3% of the weighted indicators, and in severe poverty if they are deprived in 50–100% of the weighted indicators.

### Microdata sources

The global MPI is computed using multi-topic household surveys, with the data collection supported primarily by DHS and MICS. For few countries, data from national household surveys that are comparable to DHS and MICS are used. Household surveys are required to meet several criteria for inclusion in the global MPI estimations. First, the household surveys must be representative, at least at the national level. Second, the microdata and related documentation (e.g., questionnaires and survey reports) must be available, either directly or upon e-registration.

In some cases, a single missing survey item results in the exclusion of the entire country, despite all remaining items being available. To navigate this trade-off between comparability and country coverage, a third requirement is that at least one of the health indicators (child mortality and nutrition) and at least one of the education indicators (years of school and school attendance) can be computed from the survey. If some deprivation indicators are missing, the other indicator weights in the affected dimension are increased so that the dimensional weights remain unchanged. Estimates based on data with missing deprivation indicators are flagged throughout. This approach allows researchers to decide on a case-by-case basis whether a particular analysis is meaningful. Finally, household surveys published by DHS and MICS are prioritised to achieve highly comparable estimates; household surveys by national agencies or statistical offices are only considered in the absence of DHS or MICS surveys. Table 2 provides an overview of the underlying microdata. For each country, the table shows the period of observation covered, the number of surveys used, the type of survey, the number of available subnational regions, and whether the country lacks a particular indicator. Using this information the underlying microdata sets can also be identified on the websites of the DHS (https://dhsprogram.com/data/available-datasets.cfm) and the MICS (https://mics.unicef.org/surveys), respectively. For further details, on the microdata, see also the respective methodological note for each release^32–34^.

**Table 2:** Information on underlying microdata samples.

Country code	First year	Last year	# years	Survey names	# regions	Missing indicator
Afghanistan	2010	2016	2	DHS MICS	8	Nutrition
Albania	2008	2018	2	DHS	12	
Algeria	2012	2019	2	MICS	7	
Armenia	2010	2016	2	DHS	—	
Bangladesh	2014	2019	2	DHS MICS	7	
Belize	2011	2016	2	MICS	7	
Benin	2014	2018	2	DHS MICS	12	
Bolivia	2003	2016	3	DHS EDSA	9	
Bosnia and Herzegovina	2006	2012	2	MICS	—	Child mortality
Burkina Faso	2006	2010	2	DHS MICS	—	
Burundi	2010	2017	2	DHS	5	
Cambodia	2010	2022	3	DHS	19	
Cameroon	2011	2018	3	DHS MICS	12	
Central African Republic	2000	2019	3	MICS	8	
Chad	2010	2019	3	DHS MICS	20	
China	2010	2014	2	CFPS	3	Housing
Colombia	2010	2016	2	DHS	16	Nutrition
Congo	2005	2015	2	DHS MICS	4	
Congo, Democratic Republic of the	2007	2018	3	DHS MICS	11	
Côte d’Ivoire	2011	2016	2	DHS MICS	11	
Dominican Republic	2007	2019	3	DHS MICS	10	Nutrition
Ecuador	2013	2018	2	ECV ENSANUT	24	
Egypt	2008	2014	2	DHS	23	Cooking fuel
eSwatini	2010	2014	2	MICS	4	
Ethiopia	2011	2019	3	DHS	11	
Gabon	2000	2012	2	DHS	5	
Gambia	2005	2020	4	DHS MICS	8	
Ghana	2011	2018	3	DHS MICS	10	
Guinea	2012	2018	3	DHS MICS	8	
Guinea-Bissau	2014	2019	2	MICS	9	
Guyana	2009	2020	3	DHS MICS	2	
Haiti	2012	2017	2	DHS	10	
Honduras	2005	2019	3	DHS MICS	16	Electricity
India	2005	2021	3	DHS	29	
Indonesia	2012	2017	2	DHS	33	Nutrition
Iraq	2011	2018	2	MICS	18	
Jordan	2012	2018	2	DHS	12	
Kazakhstan	2010	2015	2	MICS	16	
Kenya	2008	2014	2	DHS	8	
Kyrgyzstan	2005	2018	3	MICS	8	
Lao PDR	2011	2017	2	MICS	17	
Lesotho	2009	2018	3	DHS MICS	4	Cooking fuel
Liberia	2007	2020	3	DHS	5	
Madagascar	2008	2021	3	DHS MICS	22	
Malawi	2010	2020	3	DHS MICS	27	
Mali	2006	2018	3	DHS MICS	9	
Mauritania	2011	2021	3	DHS MICS	12	
Mexico	2012	2021	4	ENSANUT	4	Child mortality
Moldova	2005	2012	2	DHS MICS	4	
Mongolia	2010	2018	3	MICS	5	
Montenegro	2013	2018	2	MICS	3	
Morocco	2011	2018	2	PAPFAM	—	
Mozambique	2003	2011	2	DHS	11	
Namibia	2006	2013	2	DHS	13	
Nepal	2011	2019	3	DHS MICS	7	
Nicaragua	2001	2012	2	DHS	17	
Niger	2006	2012	2	DHS	8	
Nigeria	2013	2021	4	DHS MICS	37	Nutrition
North Macedonia	2005	2019	3	MICS	8	Child mortality
Pakistan	2012	2018	2	DHS	6	
Palestine, State of	2010	2020	3	MICS	2	
Peru	2012	2021	4	DHS ENDES	25	
Philippines	2013	2017	2	DHS	17	Nutrition & School attendance
Rwanda	2010	2020	3	DHS	5	
Sao Tome and Principe	2008	2019	3	DHS MICS	4	
Senegal	2005	2019	3	DHS	11	
Serbia	2010	2019	3	MICS	4	
Sierra Leone	2013	2019	3	DHS MICS	4	
Sudan	2010	2014	2	MICS	–	
Suriname	2006	2018	3	MICS	5	Child mortality
Tajikistan	2012	2017	2	DHS	5	
Tanzania	2010	2016	2	DHS	8	
Thailand	2012	2019	3	MICS	6	
Timor-Leste	2009	2016	2	DHS	13	
Togo	2010	2017	3	DHS MICS	6	
Trinidad and Tobago	2006	2011	2	MICS	5	Nutrition
Tunisia	2011	2018	2	MICS	7	
Turkmenistan	2006	2019	3	MICS	6	Cooking fuel
Uganda	2011	2016	2	DHS	4	
Ukraine	2007	2012	2	DHS MICS	5	Nutrition
Viet Nam	2013	2021	2	MICS	6	Nutrition
Yemen	2006	2013	2	DHS MICS	–	Nutrition
Zambia	2007	2018	3	DHS	9	
Zimbabwe	2010	2019	3	DHS MICS	10	

### Indicator harmonisation over time

In survey data, it is common for questions and items to vary slightly over time. In our data, we observe, in particular, changes in the (i) eligible population (e.g., the age groups for which nutrition data is collected) and (ii) survey item availability (e.g., data on computer availability are not collected in all surveys). In order to harmonise the deprivation indicators over time, we restrict the eligible population and the available survey items as needed to the common domain. Below, we briefly explain the most common changes in deprivation indicators due to harmonisation over time. For further details on which indicator was harmonised in which way for every country, see the respective methodological note for each release^32–34^.

First, the harmonised nutrition indicator often relies only on the anthropometric information from children under five years of age, since related information for adults was not collected for all years. Similarly, we ignored the nutritional information of adult males if nutritional information was only provided in all years for adult women and children. The harmonised child mortality indicator may ignore birth history information about the age of the child and the year of its death (see Table 1) if at least one survey lacks this information. The harmonised child mortality indicator then considers any child who has died in the household, independently of when the death occurred. The drinking water indicator relies on information about both the source and the round-trip time to obtain the water, while the sanitation indicator uses both the type of toilet and whether it is shared with other households. If information about the round-trip time or whether the toilet is shared is not collected, the harmonised indicators rely only on the source of drinking water or the type of toilet, respectively. A household is usually considered deprived in housing if the floor, roof, or walls are constructed using poor materials. If any of the materials are missing in one of the surveys (e.g., wall materials), the harmonised deprivation indicator relies on the remaining comparable materials (i.e., floor and roof, in this case). Finally, the assets indicator relies on the ownership of a car or truck and eight smaller asset items, such as a television or bicycle (see Table 1 for details). While earlier surveys may not have collected information about mobile phones or computers, more recent surveys may lack data on landline telephones or animal carts. The harmonised assets indicator includes only ownership of items that are consistently available across all survey periods and relevant for the global MPI.

### Missing values

Missing values are a common issue in survey datasets. For the global MPI estimates, we address missing values in several ways. First, in some cases we construct a particular deprivation indicator despite missing responses following the approach to provide a lower-bound estimate for those deprivations. Specifically, we consider the household to be non-deprived if we have at least partial information suggesting that a household may not be deprived, and in particular when this presumption is supported by further complementary information. For example, in the absence of child mortality information from any eligible women, if a man in the same household reported no child mortality, then the entire household is considered as non-deprived in child mortality.

The years of schooling indicator is defined as missing if information for at least two-thirds of the eligible household members is missing while the remaining household members all report less than six years of education. The school attendance indicator is defined as missing if information for two-thirds of the school-age eligible children is missing while the remaining eligible children are attending school. The motivation for doing so is the lack of evidence to conclusively construct the deprivation indicator. Specifically, for considering a household as non-deprived in years of schooling, it is sufficient to observe at least one member with more than six years of schooling. Conversely, for considering a household as deprived in school attendance it is sufficient to observe at least one eligible child not to attend school.

Across the indicators of living standards, if a household uses an improved source of drinking water but the round-trip time to the water source is missing, then the household is identified as non-deprived by the source. For the sanitation indicator, if a household has access to an improved toilet facility but information on whether it is a shared facility is missing, then the household is identified as non-deprived by type of facility. Note that this approach results in a lower-bound estimation for a particular deprivation.

Second, in our estimations we can consider only those households for which we can construct all deprivation indicators; observations with missing deprivation indicators are dropped (case-wise deletion). Since excessive sample drop would bias our estimates, we first report the proportion of missing values individually for every deprivation indicator and also the retained sample, which we can ultimately use in our estimation after accounting for missing values in all deprivation indicators. In some cases, we consider the sample drop to be highly problematic and thus refrain from reporting certain estimates. Specifically, we omit subnational estimates if the sample drop exceeds 15% at the national level or 25% at the subnational level.

### Estimation

In this section, we present selected aspects related to the estimation procedures of the global MPI. First, the entire production of the global MPI, including both data cleaning and deprivation indicator construction and estimation, is carried out using Stata 17. Moreover, the sampling of most datasets relies on complex survey designs, with stratified two-stage clustered sampling being the most common form. The microdata variables that contain information about the survey design (psu, stratum, hhweight) are coded such that we can rely on Stata’s svyset command to account for the complex survey design. Accordingly, all estimates rely on the survey weights as provided by the data distributor. Specifically,


svyset psu [pw = weight], strata(strata) singleunit(centered)


All deprivation indicators in the cleaned microdata begin with d_ followed by their abbreviated name and _01, which identifies the harmonised indicator (e.g., d_cm_01 for the harmonised child mortality indicator). The estimations rely on the user-written Stata package mpitb (version 0.4) which is publicly available in the Statistical Software Component (SSC) archive and GitLab and documented in a companion paper^35^. The estimation proceeds with a two-step procedure: first, we specify the indicators of the MPI of interest, then we choose the (sub)indices to be estimated and the underlying parameters, among other things. Most of the estimates for a single country provided in this database can be obtained using the following two commands:


mpitb set, d1(d_cm_01 d_nutr_01, n(hl)) d2(d_satt_01 d_educ_01, n(ed)) ///d3(d_elct_01 d_sani_01 d_wtr_01 d_hsg_01 d_asst_01 d_ckfl_01, name(ls)) n(gmpi_hot)



mpitb est, k(33) w(equal) n(gmpi_hot) tv(t) m(all) indm(all) aux(all) ///svy lfr(mylevs, replace) over(region_01 area agec2 agec4) dou ///cotm(all) coty(year_cot) coto(inseq nor) cotfr(mycots, replace)


The commands as shown above do not account in any particular way for item or unit non-responses (e.g., multiple imputation, adjustment of sampling weights). Instead, observations that exhibit a missing value in a relevant variable, such as deprivation indicators, disaggregation, or survey design variables, are removed from the estimation sample (case-wise deletion). Non-response rates are, however, monitored and reported in this database.

The household surveys used for the estimation of the global MPI are representative at the national or subnational level and usually also for the urban and rural areas in a country. For most countries, therefore, we also provide estimates by subgroups to understand whether poverty is increasing or decreasing across subgroups. For example, in terms of age group, we may find that poverty fell more slowly among children living in MPI poor households than among adults in similar households. In terms of area, results may indicate significant decrease in poverty in rural areas, but there was no significant reduction in the incidence of poverty in urban areas. Since for most countries the samples are also representative at lower administrative levels (e.g., governorates, provinces, regions, states, or zones), we also provide related estimates where possible. If necessary, subnational regions are carefully harmonised to ensure comparability over time; where it is impossible to attain comparability, regions are excluded from the analysis^32^.

Finally, the estimation of annualised change rates requires the difference between two survey periods. For surveys that are fielded across two or more periods, the analysis takes the average of the survey periods for calculating annualised change. Taking the average across the survey periods means we usually operating with a lower bound of possible absolute annual reduction compared with counting the mean or median of the month and year of the interviews to produce the annualised change^34^.

### Summary of policies

We close this section with a summary of the policies mentioned in the previous subsections. Estimating and publishing data such as the global MPI requires various decisions that go beyond those inherent to multidimensional poverty measurement (e.g., deprivation indicators and their thresholds and weights). Usually, decisions whether to include a particular estimate involve trade-offs (e.g., a higher country coverage versus perfectly comparable indicator construction). Related policies describe the rules of how these inescapable trade-offs are navigated and how related decisions are made. The documentation of such policies is essential for both the transparency of the computations and the understanding of the reach and limits of comparability. Naturally, these related policies are not fixed forever and may be subject to revision at some point. However, doing so would require a good reason and a comprehensive and balanced assessment. Compliance with the revised policy should also be anticipated. Major policies underlying the production of this database include the following.

1. The survey data must be representative, at least at the national level, easy to access, and well-documented.
2. The survey data must permit the construction of at least one health indicator and one education indicator. For missing deprivation indicators, the indicator weights will be increased such that the dimensional weights remain unchanged.
3. The deprivation indicator construction relies only on usual household members (and ignores non-usual household members).
4. Households with no eligible members (e.g., school-age children for the school attendance indicator) are assumed to be non-deprived in this particular indicator.
5. We do not publish subnational estimates for a particular country if (i) the survey does not permit analyses for subnational regions, (ii) a harmonisation of the region variable over time is not straightforward, or (iii) the sample drop exceeds 15% at the national or 25% at the subnational level.
6. For harmonised deprivation indicators over time, we restrict (i) the eligible population and (ii) the available survey items to the common domain. The indicator harmonisation principle may be revised in the future as the number of survey time points for a given country increases.

## Data Records

### Release content and repository

The global MPI is released on an annual basis. As new survey datasets become available, related estimates are added to the database. Older survey datasets are added where resources and indicator harmonisation permit. Consequently, newer releases of the database may also contain new estimates for more distant periods. To ensure transparent and replicable downstream analyses of the global MPI data, every release is separately archived and documented by its own methodological note, which records the related decisions. Releases are versioned; the estimates published in 2021 are version 1, the estimates underlying this paper and published in 2023, are version 3^8^. Previous versions are documented in the respective methodological notes^33^,^34^. Each release can also be identified by its own DOI. The data, the methodological notes, and the database licence (CC-BY) are available from the OPHI website and the Oxford University Research Archive (ORA)^8^.

### Result files

The data of the global MPI is distributed in two separate files: one for the level estimates and one for the change estimates. Both files are available in different formats, including the Stata format (dta) and comma-separated values (csv). The structure of both files follows from the key design principle that (i) each row (each observation in Stata) refers to a single estimate and (ii) each estimate has a nucleus (comprising the point estimate, its standard error, etc.) and meta-information which allows the content of an estimate (the estimated measure, the country, etc.) to be identified. Table 3 shows some data entries for illustration purposes. Specifically, the table shows six point estimates (b) with their standard errors (se), and the remaining variables provide further details about the estimates (headcount ratio and intensity for India at the national level in three different years for the preferred poverty cutoff and weighting structure).

**Table 3:** Example data entries.

ccty	year	survey	measure	b	se	k	wgt	loa
IND	2005-2006	DHS	A	51.33	0.19	33	equal	nat
IND	2005-2006	DHS	H	55.07	0.43	33	equal	nat
IND	2015-2016	DHS	A	43.96	0.06	33	equal	nat
IND	2015-2016	DHS	H	27.68	0.16	33	equal	nat
IND	2019-2021	DHS	A	41.98	0.06	33	equal	nat
IND	2019-2021	DHS	H	16.39	0.12	33	equal	nat

Table 4 provides further details about the variables available in the results files. Most variables never have missing values as they describe the content of a particular entry, including the country codes (ccty and ccnum), name and year of survey (survey and year), the level of analysis (loa), and the measure estimated (measure). The level of analyses distributed in the database includes national and regional (i.e., subnational) estimates by area (urban and rural) and by age group. Table 4 also indicates those variables which may not apply to all estimates and thus may have missing values. For instance, the subgroup identifier subg is only needed for disaggregations and, consequently, is missing for national-level estimates. Likewise, the variable indicator is missing for measures which are not indicator-specific, and the variables k and wgts, which contain the values of underlying parameters (poverty cutoff and weighting scheme), are missing for population share estimates, for example.

**Table 4:** Main variables of the results files.

Variable	Level file	Change file	Missing allowed	Description
ccty	•	•		ISO-country code
ccnum	•	•		numeric country code
survey	•	•		name of survey (e.g., DHS)
year	•	•		year of survey
ctype	•	•		0 = level, 1 = absolute change, 2 = relative change
loa	•	•		level of analysis, is one of nation, region, area, or agegroup
measure	•	•		measure estimated
b	•	•		point estimate
se	•	•	◦	standard error
ll	•	•	◦	lower bound of confidence interval
ul	•	•	◦	upper bound of confidence interval
tval	•	•	◦	$t$-value for the null of the coefficient being zero
subg	•	•	•	numeric group identifier within loa (if applicable)
k	•	•	•	poverty cutoff (if applicable)
wgts	•	•	•	weighting scheme (if applicable)
indicator	•	•	•	name of indicator (if applicable)
misind	•	•	•	name of missing indicator (if applicable)
t0	—	•		first period of change ($t\mathrm{=1,2,}\ldots ,T$)
t1	—	•		second period of change
year0	—	•		year of first period of change
year1	—	•		year of second period of change
ann	—	•		dummy for annualised change; missing for levels

^•^ indicates whether variable exists in level and change file, respectively and whether values may be missing; ^◦^ indicates that variables may be missing for selected measures.

Table 4 also points to the main difference between the level file and change file. The estimate of a change essentially features the same variables as the estimate of a level. A change estimate is also characterised by (i) the beginning and end periods and (ii) whether it is annualised or raw. Consequently, the change file contains two variables, t0 and t1, which contain the values of the country-specific time integer for the beginning and end periods, and two variables, year0 and year1, which contain the years of the surveys underlying each change estimate. Finally, the variable ann indicates whether the changes are raw or annualised.

In addition to the variables shown in Table 4, the database includes convenience variables which facilitate downstream analysis, including easier-to-read labels. All these variables carry the suffix _lab in their name. For instance, cty_lab contains the full country name instead of the ISO country code, and ind_lab contains the full names of deprivation indicators instead of cryptic abbreviations. Particularly useful for downstream analysis are the variables region_lab, area_lab, agec2_lab, and agec4_lab, which are only defined for the indicated level of analysis and contain an easy-to-read label for the respective subgroup such as subnational regions. Further labelling variables include misind_lab, dim_lab, and measure_lab.

Table 5 provides an overview of the measures available in the results files. First, the database provides estimates for the key measures of the Alkire–Foster framework, including the MPI (adjusted headcount ratio) itself, several partial indices (e.g., the headcount ratio, the intensity, and censored headcount ratios), but also uncensored headcount ratios and population shares. Besides the quantities of the Alkire–Foster framework, the database also provides some auxiliary measures. For instance, N contains the number of observations of the estimation sample. The variables mv_w and mv_uw both provide information on missing values and retained samples, either using or ignoring survey weights. Where indicator is not missing, mv_w and mv_uw contain the proportion of missing values for that particular indicator. Where indicator is missing, mv_w and mv_uw report the retained samples. Finally, the database also contains the severity (sev) and vulnerability vuln measures. Severity reports the proportion of people who are severely poor, which means they suffer from 50% or more of the maximum possible deprivations. Put differently, severity is the headcount ratio for poverty cutoff $k=\mathrm{50 \% }$. Vulnerability shows the proportion of people with a deprivation score of 20% or more but less than 33% (people who are close to being poor).

**Table 5:** Measures available in the database.

Measure	Description	Indicator-specific	Comment
M0	MPI (adjusted headcount ratio)		*H* × *A*
H	headcount ratio		proportion of population which is poor
A	intensity		average deprivation among poor people
hd	uncensored deprivation rate	•	proportion of population which is deprived in indicator
hdk	censored deprivation rate	•	proportion of population which is poor and deprived in indicator
popsh	population share		population share of particular subgroup (if applicable)
N	number of observations		number of observations in estimation sample
mv_uw	missing values (unweighted)	•	proportion of missing values for indicator / overall retained sample (unweighted)
mv_w	missing values (weighted)	•	proportion of missing values for indicator / overall retained sample (weighted)
sev	severity		proportion of population which is severely poor
vuln	vulnerability		proportion of population which is vulnerable

The results files of this database are comprehensive in the sense that they contain all data which is used in other outlets of OPHI such as the conventionally produced spreadsheets available on OPHI website (in particular, the spreadsheet ‘Trends Over Time’) or the respective findings in the country briefings.

## Technical Validation

In this section, we present selected quality checks for the estimates of this database, which are implemented at different stages. A first set of quality checks is undertaken for selected deprivation indicators whose construction are similar to those produced in the survey reports published by DHS and MICS and their national collaborators, which are renowned providers of high-quality survey data. Their survey reports incorporate cross-tabulations for a wealth of indicators^36,37^, which facilitates the quality checks for all living standard indicators of the global MPI. In particular, we compare for our estimates and the survey report, the proportion of (1) households with electricity; (2) household members with improved sanitation; (3) household members with improved access to safe drinking water; (4) households with rudimentary and finished floor materials; (5) households with finished construction materials for the walls and roof; (6) households using clean fuels and technologies for cooking; (7) households that own a television, radio, telephone, refrigerator, bicycle, motorbike, computer, animal cart, and car or truck. We also compare selected demographic figures, including the proportions of (1) urban and rural population; (2) population living in each sampled subnational region; (3) households successfully interviewed; (4) children under five years old successfully measured; (5) women in the reproductive age group (15–49 years) successfully interviewed; and (6) men successfully interviewed (for surveys that implemented a male questionnaire). Continuous communication is maintained with teams in DHS, MICS, and other data providers to resolve any mismatches between our tabulations and those provided in survey reports.

In addition, we carefully monitor and report missing values in all our deprivation indicators, as discussed above. Specifically, we report the share of missing values for individual deprivation indicators, as well as the sample that is ultimately retained. This documentation of missing values allows researchers to make a critical assessment of the issue for their particular purpose. Finally, in our estimation and production routines, we also rely on certification scripts, which perform quality checks against the microdata to detect potential mistakes or issues (e.g., whether all deprivation indicators are 0–1 coded).

## Usage Notes

### Result files

We present seven brief remarks on the usage of the result files. First, since the data is provided in csv format, all statistical software packages are able to process these files. However, specialist packages such as Stata or R, which can easily manage larger datasets, are preferable. Together, the level and change estimates for the 2023 release of this database amount to more than 350,000 observations across the result files.

Second, in order to facilitate merging additional data, result files include (i) the ISO country code (alpha-3 and numeric) and (ii) the year of the survey as stated by the data provider, which allows users to adopt the most appropriate approach in their use case (e.g., how to choose years from surveys spanning several years). Unfortunately, no universal ISO code is available for subnational regions. Users can, however, easily generate their own custom identifier variables. For example, the id variable generated by the code below, identifies each subgroup of every level of analysis, including all subnational regions, among others. The subsequent distinct, a user-written convenience tool^38^, confirms that the database contains 814 subnational regions.


gen id = ccty + "_" + loa + "_" + strofreal(subg) distinct id if loa == "region"


Third, a convenient strategy for retrieving the desired estimates is to first choose how to display or plot the nucleus of an estimate, for example, the point estimate and standard error (variables b and se), and then select the content of an estimate based on the metadata, for example, the entity of an estimate, the underlying parameters, etc. In Stata, the estimates of interest can be conveniently selected using if conditions. For instance, the data shown in Table 3 above can be retrieved using


li ccty year survey measure b se k wgt loa ///if ccty == “IND” & inlist(measure,”H”,”A”) & loa == “nat”


**Fig. 1:**
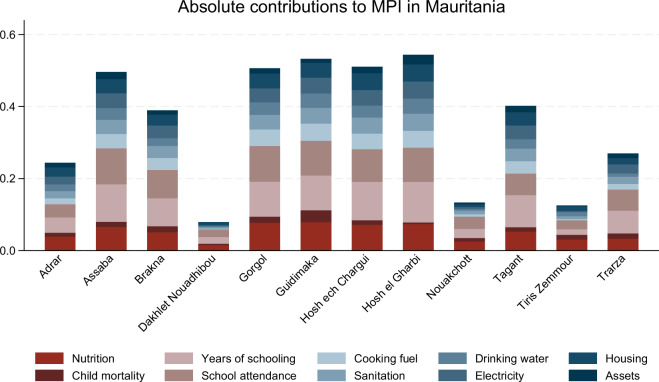
Absolute indicator contributions in Mauritania (survey 2011). For the underlying Stata command, see the main text.


Fourth, as previously mentioned, the results files contain several convenience variables that facilitate the downstream analysis; these are suffixed by _lab. As well as containing detailed labels, the advantage of variables such as region_lab is that they are only defined for the respective level of analysis (region in this case), resulting in shorter and simpler commands for the analysis. For example, to plot a stacked bar chart of the absolute indicator contributions for all subnational regions in Mauritania for the first period of observation, one can simply issue the following command in Stata (for the related output, see Fig. 1):

graph bar b if ccty == "MRT" & t == 1 & measure == "actb", ///over(ind_lab) over(region_lab) stack asyvar leg(rows(2) colf) ///yti("") ti(Absolute contributions to MPI in Bangladesh)


**Fig. 2:**
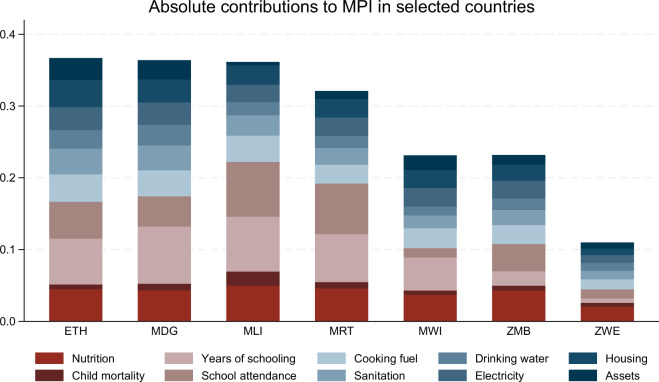
Absolute indicator contributions for selected countries in Sub-Saharn Africa. For the underlying Stata command, see the main text.


Fifth, adapting such graphing commands to other contexts, requires only minor modifications. For example, plotting the stacked bar chart of the absolute indicator contributions for selected countries in Sub-Saharan Africa, as shown in Fig. 2, can be achieved using the Stata commands below. The generated variable T helps to restrict the analysis to the most recent survey year for each country.

bys ccty: egen T = max(t)graph bar (asis) b if inlist(ccty, "MRT","ZMB","ZWE","MDG","ETH","MWI","MLI") ///& t == T & measure == "actb" & loa == "nat", ///over(ind_lab) over(ccty) stack asyvar ylab(,format(yti("") ti(Absolute contributions to MPI in selected countries)


**Fig. 3:**
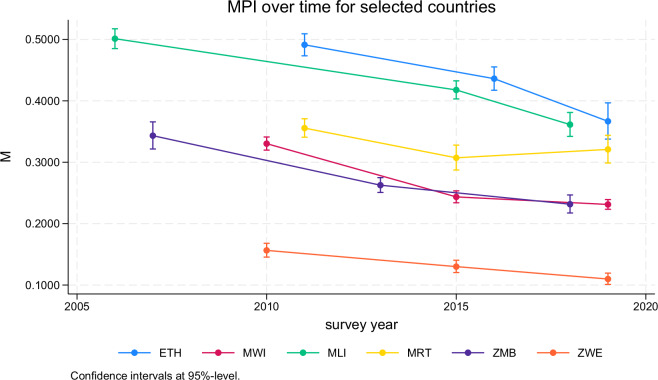
Adjusted headcount ratio (M or MPI) over time for selected countries in Sub-Saharn Africa. For the underlying Stata command, see the main text.


Sixth, as the result files also provide standard errors and confidence intervals for many point estimates, they can be easily added to graphs or tables, too. Figure 3, for instance, shows the level estimates for the adjusted headcount ratio (*M*) for selected countries of Sub-Saharan Africa over time. The underlying code is as follows:

use GMPI_HOT_2023_puf.dta if loa == "nat" & measure == "M0", cleardecode year, gen(year_str) maxl(4)destring year_str, gen(year_num)sort year_numtw (rcap ll ul y if ccty == "ETH", col(stc1))(con b y if ccty == "ETH", col(stc1)) ///(rcap ll ul y if ccty == "MWI", col(stc2))(con b y if ccty == "MWI", col(stc2)) ///(rcap ll ul y if ccty == "MLI", col(stc3))(con b y if ccty == "MLI", col(stc3)) ///(rcap ll ul y if ccty == "MRT", col(stc4))(con b y if ccty == "MRT", col(stc4)) ///(rcap ll ul y if ccty == "ZMB", col(stc5))(con b y if ccty == "ZMB", col(stc5)) ///(rcap ll ul y if ccty == "ZWE", col(stc6))(con b y if ccty == "ZWE", col(stc6)) ///if measure == "M0", yti(M) ti(MPI over time for selected countries) ///leg(order(2 4 6 8 10 12) lab(2 "ETH") lab(4 "MWI") lab(6 "MLI") ///lab(8 "MRT") lab(10 "ZMB") lab(12 "ZWE") rows(1) pos(6)) ///note("Confidence intervals at 95%-level.")


Finally, one can also combine the change and level files for certain analyses. For example, suppose that we would like to see absolute reductions in the adjusted headcount ratio ($M$) conditional on the level of the initial period for all subnational regions of a particular country. First, we load the level estimates of the adjusted headcount ratio for all subnational regions and all periods of observation. Then, we create a variable t0 = t which will allow us to merge level estimates of the initial period into a particular change estimate. Now we can load the absolute changes of the adjusted headcount ratio for all subnational regions.


use GMPI_HOT_2023_puf.dta if loa == "region" & measure == "M0", cleargen t0 = tframe put *, into(mylev)use GMPI_COT_2023_puf.dta if loa == "region" & measure == "M0" & ctype == 1, clearfrlink m:1 ccty subg t0, frame(mylev)frget lt0 = b, from(mylev)format b lt0 %9.3f


After linking the frames and copying the variable of interest, we can plot our graph using twoway.


tw (sca b lt0, ml(region_lab)) if loa == "region" & ccty == "MLI" & t0 == 2, ///xsca(alt) xlab(0 (.1) .7) ylab(-0.03 (.005) 0.005) yline(0) ///leg(off) xti("M (level in initial period)") yti({&Delta}M)


**Fig. 4:**
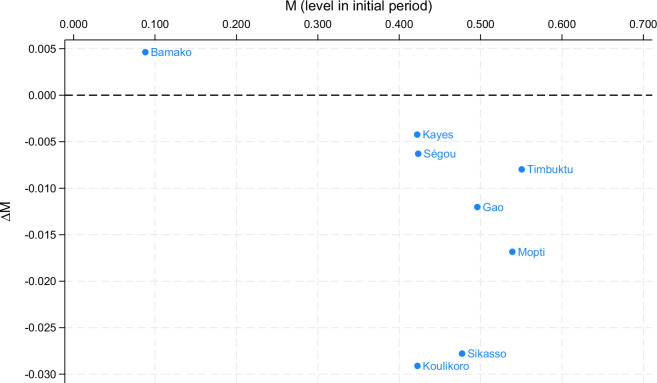
Absolute reductions of the adjusted headcount ratio for subnational regions in Mali in 2015–2018. For the underlying Stata command, see the main text.

Note that the graphing command above requires only minor modifications to produce the same figure for a different country (e.g., ccty == "MRT"), a different initial period and change (e.g., t0 == 1), or a different measure (e.g., measure == "H") that is available in the database. Naturally, there are various ways to improve Fig. 4, such as plotting labels for only selected regions, drawing symbol markers proportional to population shares, automatically computing ranges for labels, etc.

### Microdata

The underlying microdata sets of most countries (largely from DHS and MICS) are available only for research purposes and therefore cannot be publicly shared. In order to construct the deprivation indicators underlying the estimates of this database, one has to run one do-file for every survey (211 do-files in total, all of which are available in the ORA repository^8^ and on the OPHI website). These do-files also rely on other user-written Stata programs (e.g., who2007, mdesc). Therefore, analyses using the microdata underlying the global MPI are also feasible.

## Data Availability

Stata (version 17) was used for both data preparation and estimation. The Stata scripts (do-files) for data cleaning and deprivation indicator construction are available for this release from the ORA repository^8^ and the OPHI website. The estimation was carried out with the user-written Stata package mpitb (version 0.4), which is available from the Statistical Software Components (SSC) archive and GitLab^35^. The mpitb package is distributed under an MIT licence. The key commands for the global MPI estimation of a particular country are presented above.
